# Boredom as the originator of a desideratum - reflections on the creative and suppressive consequences of boredom in the school context

**DOI:** 10.3389/fsoc.2023.1214069

**Published:** 2023-08-24

**Authors:** Anke Zeißig

**Affiliations:** Department of Educational Psychology, Institute of Health Science, Faculty of Social Work, Health and Music, Brandenburg University of Technology Cottbus-Senftenberg, Cottbus, Germany

**Keywords:** boredom, creativity, education, school, spirituality

## Abstract

Bored children begin to draw, do crafts, to fidget - or they do something bad. Others fall silent, withdraw, or become lethargic. Research on school-related boredom has focused primarily on the negative consequences of boredom, such as decreased cognitive performance, motivation or attentativeness, or disruptiveness. These negative aspects of boredom can be contrasted by the notion that boredom can promote creative performance. This paper reflects on boredom's creative and suppressive consequences as an interplay of personality traits and behavioral possibilities in school situations, on the one hand, and as an interplay of situational experiences with constituent developmental processes on the other. It is proposed that boredom is a gauge of the learner's resonance with school content, learning and/or developmental relationships. Boredom indicates a psychological need and its desideratum. Thus, both creative and suppressive potentials are inherent in boredom.

## 1. Introduction

Is boredom good or bad, does it lead to creativity or lethargy, does it make us productive or does it inhibit us? In this regard boredom seems to be a paradox. In the seventeenth century, Blaise Pascal wrote “Ainsi, sans la faim des choses spirituelles, on s'en ennuie” (Pascal, 1873, p.391), which translates as: without the hunger for spiritual or mental things, one becomes bored. In contrast, Herrero-Puertas (2021) questions whether boredom should be thought of as an “empty stomach” of the mind. Thus, this also seems to be a paradox: that we are bored without spiritual hunger and we are bored because we are mentally hungry. However, these two different metaphors point to essential questions for empirical research on school-related boredom, namely: how do our children learn what they are spiritually or mentally hungry for - and how can this hunger be satiated in school? Thus, the focus is directed, on the one hand, to the long-term formative, person- and development-related aspects and, on the other hand, to situational, social, or pedagogical factors.

Boredom is considered an interactional phenomenon that is socially acquired (Thoits, 1989; Brissett and Snow, 1993; Neyer and Lehnart, 2015; Ohlmeier et al., 2020). Social relationships provide the defining context for personality development (Caspi, 2000). Thus, boredom has an impact on the personality development of students (Pekrun, 2016). Learning and performance-relevant behaviors are the product of an interaction of an individual's character and experience with a particular social learning environment and its situational stimuli (Kärner et al., 2017; Kärner and Sembill, 2022). Thus, the personality of children and adolescents influences the learning process, which in turn influences social relationships (Neyer and Lehnart, 2015). The learning individual is in reciprocal interaction with the learning environment in which it interacts (Bandura, 1978). Although this paper is based on the view of a dynamic-interactionist paradigm (Asendorpf, 1996), i will discuss the two aspects – person-related and situation-related aspects of boredom – separately.

The distinction between these two different aspects and how they may change over time is essential for the empirical study of boredom and could offer a way to differentiate between the creative or suppressive consequences of boredom for children and adolescents. In research, the cumulative effect of situations on the development of basic psychological functioning and personality (Baltes, 1990; Baltes and Smith, 2004) can be studied separately from the effect of personality traits on children's behavior (Nave et al., 2010) in school. This work will take both perspectives into consideration intending to distinguish the suppressive potential of boredom for the development of children and adolescents from its creative function.

## 2. Person-related causes of boredom and its creative and/or suppressive consequences

Transactions take place between individuals' personalities and social relations, forming relatively stable patterns of interaction (Neyer and Lehnart, 2015; Asendorpf et al., 2017; Asendorpf and Motti-Stefanidi, 2018). The tendency, stable over time, to experience more boredom than others is referred to as boredom proneness and is characterized by an inability to experience internal stimulation (Farmer and Sundberg, 1986). This tendency to boredom, for example, inhibits the persistence with which tasks are performed and reduces the ability to engage in activities (Vodanovich and Kass, 1990). However, the occurrence of boredom is also understood as a state of simultaneous impulsivity and tension (Fenichel, 1953) that requires a regulation of interest or meaning processes (Elpidorou, 2018). Temperamental tendencies, such as behavioral inhibition and frustration tolerance, or personality traits, such as conscientiousness and impulsivity, are relevant to such regulatory processes (Calkins and Fox, 2002; Hoyle, 2006). To buffer boredom, temperamentally disinhibited adolescents in particular exhibit delinquent behavior, but, shy adolescents tend to withdraw into themselves, (Spaeth et al., 2015). Physical activity, daydreaming, or exploratory and creative behavior can also be strategies to bring about a change in the situation or the experience of the situation (Smith, 1981; Doehlemann, 1991; Csikszentmihalyi, 2000). Due to individual differences on the level of personality traits, children and adolescents are capable of coping with boredom in different ways. Person-related boredom-coping strategies can be differentiated into: behavioral approach, behavioral avoidance, cognitive approach, and cognitive avoidance (Nett et al., 2010, 2011). Accordingly, approach behavior, is described as a strategy to change and improve a boring situation, e.g., by asking an interesting question in class, drawing a sketch to get a better understanding, interrupting a monologuing torrent of words, or asking an overtaxing lecturer for an example close to everyday life. Secretly reading under the table or scrolling through emails on the laptop during a seminar, on the other hand, would be a behavioral avoidance strategy. If a situation cannot be influenced by behavioral change, which is often the case in lessons, a reassessment of the situation can be a cognitive approach strategy and a different approach to subject matter thereby reducing boredom (Nett et al., 2010, 2011). In contrast, if students escape into dreams and mentally avoid being bored in class, they exhibit a cognitive avoidance strategy (Nett et al., 2010, 2011).

It is conceivable that these different strategies can have both constructive and inhibitory or destructive effects. A possible consequence of reading secretly under the table in class is a successful coping with boredom by bringing the child closer to an interest distant from the lesson. If this behavior is tolerated by the teacher, for example, because the lesson is not disrupted or because the teacher can allow this child this freedom as long as the other children still need time and invites him or her back to cooperate later, no conflict arises. However, the child may “learn” in this way to endure rather than change unpleasant situations, which could have long-term developmental effects. However, if the teacher perceives reading under the table as inattentive and rude and stops this behavior, he forces the child back into the boredom-producing situation. This can then become agonizing for the child, as the power structure between teacher and student in this case proves violent for the child. In this scenario, from an unpleasant emotional situation will be difficult to evade. This could cause feelings of anger but also of powerlessness and explain forms of boredom that are less distinguishable from states of apathy, depression, or a persistent inability to feel pleasure and joy (Goldberg et al., 2011). Requesting the teacher to help alleviate boredom could also have negative effects on students: Assuming that asking for additional or challenging tasks could be perceived by the teacher as criticizing the lesson, the teaching, or the teachers own person and be judged as rebellious or disruptive behavior. If negatively perceived, children would not experience a positive reinforcement of their constructive behavior and would not experience themselves as self-efficacious even though they have tried to deal with the situation creatively. Thus, how boredom is managed and whether this has a creative or suppressive effect depends very much on the particular social interaction (Brissett and Snow, 1993; Darden and Marks, 1999; Finkielsztein, 2020; Ohlmeier et al., 2020).

Personality-specific characteristics are also reflected in individual interests, and boredom is seen as a precursor to curiosity (White, 1998). Accordingly, boredom as an emotional state characterizes or signals (Elpidorou, 2018) the interplay of a person's current internal state with the current situation and is co-determined by basic personality factors, developmental aspects, as well as external framework and contextual factors (Kärner and Kögler, 2016). As a result, boredom-causing or boredom-triggering factors can differ seriously between individuals, and can control, influence, and hinder learning processes (Schiefele and Schaffner, 2006). In particular, the difference between extrinsic and intrinsic motivation, which function as regulatory styles of self-determination (Ryan, and Deci, 2000), is an important discourse in educational psychology (Schiefele and Köller, 2006). Autonomy, relatedness, and competence are considered basic psychological needs of learners within the framework of self-determination theory. The satisfaction of which is essential for the development of intrinsic motivation (Ryan and Deci, 2000). Learners who experience themselves as self-determined and competent, and who experience their environment as something they can actively shape and participate in, can act creatively (Prenzel et al., 2000; Dietrich et al., 2015). The need for autonomy or self-determination (de Charms, 1968) is considered fundamental, innate, and relevant to learning (Deci and Ryan, 1985). Freedom of action, decision-making, and design (Ulich, 1994) and frequent situations in which the individual can freely dispose of his or her time (Krapp, 1992) are considered conducive to creative processes (Herbig et al., 2008). Moreover, fostering creativity in the classroom has the potential to eliminate boredom and increase teaching effectiveness (Radeljić et al., 2020). Motivation and learning success are particularly high when tasks offer the opportunity to be creative, to develop skills, to make one's own decisions, to determine how to perform them, and are challenging, varied, non-repetitive, manageable in time, and unambiguous in task setting (Karasek and Theorell, 1990). Personality traits can thus have different effects depending on the specific circumstances and contextual conditions and can contribute to constructive, creative processes as well as have suppressive consequences.

## 3. Situational causes of boredom and its creative and/or suppressive consequences

Manifest psychological structures, which become apparent in specific situations and shape individual experiences, can be formed by the social relationships and the associated contextual and situational conditions of growing up, learning, and developing (Thoits 1989; Caspi, 2000). Such a transformation is also possible with respect to boredom and is conveyed through the interrelation of emotion and cognition (Hunter and Eastwood, 2018). The more students are in a permanent state of boredom in school, the more space boredom occupies in the psyche of the students (Yacek and Gary, 2023). This can result in a child's inability to find interest, pleasure, and joy in mental, spiritual, or cognitive activities. Djian (1994) describes this with the literary image of a class as a destroyed ghost town in which the students doze away like skeletons (Djian, 1994). In particular, findings from studies that examined the consequences of constraints on autonomy indicate that feelings of powerlessness may be responsible for chronic difficulties in making decisions, loss of motivation in school tasks, decreased physical health, and depression (Kohn, 1993). When children and adolescents feel they must do something because of external compulsion, they report higher levels of boredom than when they participate in something because of internal motivation (Caldwell et al., 1999). Further research demonstrates that a lack of support for autonomy in educational environments creates boredom (Khan et al., 2019). Learning environments are often described and perceived as places where boredom is particularly prevalent (Vogel-Walcutt et al., 2012). Boredom is a so-called performance emotion that affects learning (Pekrun, 2017) and is experienced very frequently by students (Moeller et al., 2020). According to studies, boredom significantly affects learning success (Craig et al., 2004) and is defined as an unpleasant affective state in which there is a perceived temporary lack of interest and difficulty focusing on the current activity (Fisher, 1993). As early as Lipps (1903), it was found that there is a discrepancy between the need for cognitive activity and the lack of stimulation or inability to be stimulated, resulting in boredom.

According to van Tilburg and Igou (2017), boredom can be clearly distinguished from other emotions perceived as negative, such as sadness, anger, frustration, fear, disgust, depression, guilt, shame, regret, or disappointment, by low negative valence and arousal. It is emphasized that boredom interferes with learning or the ability to acquire knowledge especially when there is a mismatch in the fit between the individual's abilities and the level of challenge provided by the task or too little choice is given to the learning process (Vogel-Walcutt et al., 2012). Smith (1981) concluded in his review that repetition, lack of novelty, and monotony in particular cause boredom and cause learners to lose interest in the subject matter (Vogel-Walcutt et al., 2012). Other aspects that have been shown in studies are that boredom is related to negative affect, off-task thoughts, overestimation of elapsed time, reduced ability to act, as well as over- and under-stimulation and activation of the default mode network (Raffaelli et al., 2018) and can be caused by a decrease in personal meaning (Finkielsztein, 2021), significance, complexity, or challenge (Elpidorou, 2018). In addition, studies indicate that students who are bored may tend to buffer this negative feeling through delinquent behavior (Dahlen et al., 2005; Spaeth et al., 2015). Even sadistic tendencies may emerge, especially when there are no alternative behavioral options (Pfattheicher et al., 2021). Furthermore, the need to permanently regulate boredom in the classroom is a high psychological demand and burden and it's neglect can have a suppressive effect on learners' development (Gagné, 1993). Yet, as learners, children are highly effective, motivated, playful, and particularly good at actively exploring learning (Gopnik, 2020). Thinking about teaching, the transmission of knowledge and values, the development of skills and abilities, what students learn and under what conditions, and how learning, mental development, and health are interrelated, has therefore been relevant for millennia. Already Plato formulated that knowledge must be absorbed into the soul when learning and therefore it should be considered which knowledge is of harm or benefit (Platon, 1925, Protagoras 314). Learning changes knowledge structures and cognitive performance, influences developments and career opportunities, and is determined by both external factors and variables (Shuell, 1986) and by the active and self-directed construction of knowledge in interactive social and situational processes (Mandl and Krause, 2001). In addition, perceived meaningfulness is relevant to the learning process (Underwood and Schulz, 1960). If learners show boredom in class, efforts to teach and learners' opportunities or interests to absorb this knowledge or actively participate in the learning process are seen as having a less-than-optimal relationship (Elpidorou, 2018). Thus, in addition to the inability to engage in a cognitive activity, there is equally a need for it and a willingness to be stimulated by the outside world (Fenichel 1953). As a so-called adaptive emotion, Bench and Lench (2013) understand boredom as an emotion that indicates the extent to which current goals are fulfilling or even not fulfilling and thus prompts the pursuit of alternative goals or signals the need to seek or turn to other goals. Barbalet (1999) goes further by stating “boredom is a defense against and corrective of meaninglessness” (p. 642). Thus, boredom may even have a protective function (Belton and Priyadharshini, 2007), against the assimilation of knowledge that is perceived as meaningless, etc., and instead promotes the seeking of mental nourishment that has personal relevance.

## 4. Boredom as a barometer for the nutritional value of the mental and spiritual content

The context of the initial quotation of this article by Blaise Pascal is that boredom results from a lack of hunger for spiritual things and distinguishes them from physical things such as eating and sleeping (Pascal, 1873). It can therefore be assumed that spirituality here stands for the intellectual and mental, fundamental, intrinsic, and meaning-giving human phenomenon that is distinct from religious expressions (Elkins et al., 1988; Utsch, 2005; von Gontard, 2012). As an experiential and intuitive quality of thought, spirituality is a psychological process with an integrative function (Hiatt, 1986). It serves the understanding of existence (Wiggermann, 2000) and interpersonal connectedness, and it manifests itself in animistic ways, especially in children (von Gontard, 2012). As a result, it motivates questions about the meaning and value of human beings and their existence in the world (Bruns et al., 2007). The state of boredom, on the other hand, is associated with the experience of meaninglessness (Barbalet, 1999; van Tilburg and Igou, 2012, 2017) or the perception of senselessness (Fahlman et al., 2009; Chan et al., 2018). When extraneous, normative, and artificial learning environments are implemented in schools that have nothing in common with learning situations in the world, these educational environments alienate children from their diverse social backgrounds, cultural and ethnic imprints, or family references (Wheldall and Glynn, 1988; Csikszentmihalyi, 2000) and cause boredom (Tolor, 1989; Belton and Priyadharshini, 2007). Non-dialogic forms of online instruction that impede direct interactions between students and instructors are also found to have a profoundly alienating effect (Hamamra and Qabaha, 2023) - a desiccation of the inner world (Revers, 1949). Accordingly, boredom may serve students to distance themselves from certain tasks or environments (Breidenstein, 2007) that are perceived as pointless or meaningless because they are not mentally nourishing. This lack of ensouled interpersonal connectedness and meaningfull experiences, and the resulting feelings of boredom functions as an engine of play and spontaneity (Revers, 1949). Thus, boredom creates a space for contemplation, processing, invention, and imagination, for exploration, for alternative social, cognitive, or emotional experiences, and motivates change (Belton, 2001; Bench and Lench, 2013; Hunte et al., 2022). For these reasons, boredom could gauge the nutritiousness of intellectual, mental, or spiritual content. It could initiate learning and creative processes that are personally fulfilling and developmentally beneficial for children and adolescents.

## 5. Boredom as the potential for development and creativity

The creative cognition approach (Martindale, 1995; Smith et al., 1995) is based on the assumptions that creativity occurs as a process of a flexible alternation between abstract-logical thinking and unconscious thinking, both involving a high level of association building (Kris, 1952; Mednick, 1962). This is accompanied by defocused attention (Mendelsohn, 1976) and by a steady but reduced activity (low arousal) of several cortex areas that are simultaneously connected and synchronized (Martindale and Hasenfus, 1978; Martindale, 1989; Fink and Benedek, 2014). This is described by Andreasen (2005) as random episodic silent thinking and is associated with increased alpha activity. The neurobiological basis for this is the default mode network a neurological system that is active when a person is sleeping, dreaming, or relaxing, or when thoughts are running unconsciously or freely and uncensored (Andreasen, 2011). Boredom has already been shown to be related to an activation of the default mode network (Danckert and Merrifield, 2016; Raffaelli et al., 2018). The random episodes of thinking that arise from moments of boredom, relaxation, or banality potentially enable the development of ideas and creative processes (Jaynes, 1990). The emergence of new qualities that can emerge unexpectedly and inexplicably in this way is referred to as emergence (Byrne, 2002). Other findings in cognitive research also point to deactivating states, such as boredom, as favoring creative thinking (Fink et al., 2011; Baird et al., 2012; Fink and Benedek, 2014). Occasionally, a positive effect of boredom on creative and creative processes, e.g., as an intellectual stimulant (Bruner, 1980) or as favoring creative thinking skills (Gasper and Middlewood, 2014; Mann and Cadman, 2014), has been empirically demonstrated. The extent to which students enter such a state, with activation of the default mode network, during periods of boredom in the classroom has not yet been explored. Nevertheless, Gibbs (2013) suggests that by interrupting busy pedagogical work, opportunities for idle time for thinking must be created to open pathways for learners into developmental and creative processes. While intelligent cognitive performance is evident and measurable in well-defined problem spaces, creative processes are necessary for poorly defined problem spaces, and it is postulated that intelligent thinking is compromised when education, does not attend to creative thinking (Welter et al., 2017). Konrad (2014) also sees creativity as an intrinsic aspect, or even the highest level, of the intentional learning process. This process is understood here as a continuum whose complexity depends on the extent to which the learning and application situations are similar or different. The higher the degree of dissimilarity between what is learned and it's possible application, i.e., the transfer distance, the more creative learners must become and be able to draw on complex systems of intellectual development to do so (McKeachie et al., 1986). However, the current body of evidence on the relationship between boredom and creativity is so far insufficient and the understanding of possible directions of action, causal relationships, and consequences for the cognitive development of children and adolescents is lacking (Zeißig et al., manuscript in preparation).^1^ Greenson (1951) distinguished two forms, different in terms of activation state, namely apathetic and agitated boredom. More recent findings (Goetz et al., 2014; Baratta and Spence, 2015) also identify different forms of boredom that differ in terms of valence and arousal (participants' self-assessment), raising the question of the functionality behind these forms of boredom (Elpidorou, 2015). On the one hand, situations that are low-stimulus and low-stress hold the possibility to escape external stimulus-response events, open up a cognitive scope and unused cognitive potential (Eastwood and Gorelik, 2019), or engage in internal processes. Interaction with the internal world is also referred to as a fruitful form of inaction (Moran, 2003). On the other hand, boredom may also be perceived as an alarm signal to avoid situations that are hostile to development and to seek productive, stimulating, or more meaningfull experiences (Elpidorou, 2015; Moynihan et al., 2017) or to address the cause of boredom. And thirdly, creative energy is an attribute of spirituality (Haase et al., 1992) and its absence is detectable by means of boredom (Pascal, 1873). Boredom, therefore, not only indicates a lack of or need for change in a situation. It also indicates a specific psychological need, e.g., for stimulation, novelty, meaning, significance, relationship, cognitive challenge, understanding, connectedness or resonance. Thus, boredom holds both the emotional impetus for change and, at the same time, the desideratum, i.e., the necessary direction to be taken in order to remedy a state of psychological deficiency. Boredom thus has inherent emergent potential for the creativity and development of children and adolescents.

## 6. Conclusion

The seemingly paradoxical metaphor - that we are bored without a hunger for spiritual things (Pascal, 1873) and we are bored because we are mentally hungry (Herrero-Puertas, 2021) make it clear that education, on the one hand, should quench children's thirst for thinking and knowledge, but, on the other hand, first develops their hunger or appetite for valuable intellectual, mental or spiritual food (Sternberg, 2003). Human experience and development, especially in childhood, are existentially dependent on experiences of resonance and are significantly shaped by experiences at school (Rosa, 2016). If no access to meaningful contact or relationship is found, boredom develops (Zeißig, 2018). In this way, however, the state of boredom also tangibly demarcates the experience from the external situation and, as it were, creates a desideratum. In this way, an organism protects itself from receiving stimuli, content, or mental nourishment that is perceived as inappropriate, irrelevant, wrong, or harmful, and makes clear the need for change. Comprehensibility, relationship to the content, to the teacher, or to fellow human beings, cognitive challenge, meaning and significance may have been lost and thus desiderated. In this way, boredom even points us in the necessary direction or quality of change. This makes boredom a condition that has both creative and suppressive aspects immanent to it at the same time.

## Author contributions

AZ conceived, drafted, wrote the manuscript, revised, and approved the submitted version.

## Footnotes

^1^Zeißig, A., Kansok-Dusche, J. Fischer, S. M., Moeller, J., and Bilz, L. (manuscript in preparation). *The Association Between Boredom and Creativity in Educational Contexts-A Scoping Review on Research Approaches and Empirical Findings*.
